# Supplementary material for: Design and Preliminary Immunogenicity Evaluation of Nipah Virus Glycoprotein G Epitope-Based Peptide Vaccine in Mice
Source: Vaccines (Basel). 2025 Apr 18;13(4):428. doi: 10.3390/vaccines13040428 (PMC12031491; doi:10.3390/vaccines13040428)
Supplement: Supplementary file 1 [file vaccines-13-00428-s001.zip › Table_S1_B_cell_epitopes.pdf]

Table S1. B cell epitopes of Nipah virus G (NiV-G) protein

Gene	No.	Amino acid position		Length	Peptide sequence
		Start	End		
G glycoprotein	1	7	43	37	KVRFENTASDKGKNPSKVIKSYYGTMDIKKINEGLLD
	2	75	81	7	RSTDNQA
	3	91	92	2	QQ
	4	95	99	5	KGLAD
	5	102	102	1	G
	6	110	110	1	S
	7	139	214	76	NENVNEKCKFTLPPLKIHECNISCPNPLPFREYKPQTEGVSNLVGLPNNIC LQKTSNQILKPKLISYTLPVVGQSG
	8	239	245	7	SCSRGVS
	9	259	262	4	GDEV
	10	273	277	5	PSNPN
	11	300	313	14	VGDPILNSTYWSGS
	12	326	331	6	NGESYN
	13	335	342	8	FALRNIEK
	14	371	404	34	VRTEFKYNDSNCPIAECQYSKPENCRLSMGIRPN
	15	420	423	4	DEEN
	16	433	433	1	D
	17	482	499	18	NTVISRPGQSQCPRFNKC
	18	530	532	3	QTA
	19	554	558	5	EDTNA
