# Supplementary material for: Design and Preliminary Immunogenicity Evaluation of Nipah Virus Glycoprotein G Epitope-Based Peptide Vaccine in Mice
Source: Vaccines (Basel). 2025 Apr 18;13(4):428. doi: 10.3390/vaccines13040428 (PMC12031491; doi:10.3390/vaccines13040428)
Supplement: Supplementary file 1 [file vaccines-13-00428-s001.zip › Table_S2_T_cell_epitopes.pdf]

Table S2. T-Cell Epitope Prediction Result of Nipah Virus glycoprotein G (NiV-G).

Gene	Group	Amino acid position		Length	Score	Peptide sequence
		Start	End			
G glycoprotein	G11	300	313	14	0.07033	VGDPILNSTYWSGS
	G17	482	499	18	-0.36204	NTVISRPGQSQCPRFNKC
	G14	371	404	34	-0.54557	VRTEFKYNDSNCPIAECQYSKPENCRLSMGIRPN
	G1	7	43	37	-0.95364	KVRFENTASDKGKNPSKVIKSYYGTMDIKKINEGLLD
	G7	139	214	76	-1.30568	NENVNEKCKFTLPPLKIHECNISCPNPLPFREYKPQTEGVSNLVGLPNNIC LQKTSNQILKPKLISYTLPVVGQSG
