# Supplementary material for: Design and Preliminary Immunogenicity Evaluation of Nipah Virus Glycoprotein G Epitope-Based Peptide Vaccine in Mice
Source: Vaccines (Basel). 2025 Apr 18;13(4):428. doi: 10.3390/vaccines13040428 (PMC12031491; doi:10.3390/vaccines13040428)
Supplement: Supplementary file 1 [file vaccines-13-00428-s001.zip › Table_S3_G_9mer_epitopes.pdf]

Table S3. Nipah virus glycoprotein G (NiV-G) 9-mer T-cell epitopes.

Amino acid position		Length	Score	B/T-cell epitope peptide sequence	Peptide 9-mer	
Start	End				Sequence	Score
300	313	14	0.07033	VGDPILNSTYWSGS	LNSTYWSGS	0.07
					VGDPILNST	0.013
					GDPILNSTY	-0.004
					PILNSTYWS	-0.008
					ILNSTYWSG	-0.044
					DPILNSTYW	-0.099
482	499	18	-0.36204	NTVISRPGQSQCPRFNKC	NTVISRPGQ	0.045
					QSQCPRFNK	0.041
					SQCPRFNKC	0
					GQSQCPRFN	-0.121
					TVISRPGQS	-0.122
					VISRPGQSQ	-0.175
					ISRPGQSQC	-0.278
					PGQSQCPRF	-0.347
					SRPGQSQCP	-0.367
					RPGQSQCPR	-0.428
371	404	34	-0.54557	VRTEFKYNDSNCPIAECQYSKPENCRLSMGIRPN	SNCPIAECQ	0.191
					DSNCPIAEC	0.15
					NCPIAECQY	0.149
					SKPENCRLS	0.077
					CPIAECQYS	0.029
					VRTEFKYND	0.017
					NDSNCPIAE	0.012
					YSKPENCRL	-0.005
					RLSMGIRPN	-0.035
					RTEFKYNDS	-0.056
					KPENCRLSM	-0.084
					YNDSNCPIA	-0.148
					PIAECQYSK	-0.148
					CRLSMGIRP	-0.167
					FKYNDSNCP	-0.179
					KYNDSNCPI	-0.199
					EFKYNDSNC	-0.203
					NCRLSMGIR	-0.214
					QYSKPENCR	-0.224
					PENCRLSMG	-0.259
					ENCRLSMGI	-0.26
					TEFKYNDSN	-0.267
					CQYSKPENC	-0.307
					ECQYSKPEN	-0.356
					IAECQYSKP	-0.404
					AECQYSKPE	-0.482
7	43	37	-0.95364	KVRFENTASDKGKNPSKVIKSYYGTMDIKKINEGLLD	KVRFENTAS	0.282
					IKKINEGLL	0.174
					KKINEGLLD	0.15
					VRFENTASD	0.105
					SYYGTMDIK	0.002
					RFENTASDK	-0.026
					DIKKINEGL	-0.059
					KSYYGTMDI	-0.071
					IKSYYGTMD	-0.099
					YYGTMDIKK	-0.114
					TMDIKKINE	-0.163
					FENTASDKG	-0.188
					VIKSYYGTM	-0.192
					MDIKKINEG	-0.205
					GTMDIKKIN	-0.212
					SKVIKSYYG	-0.224
					PSKVIKSYY	-0.244
					ENTASDKGK	-0.25
					GKNPSKVIK	-0.265

					KVIKSYYGT	-0.322
					YGTMDIKKI	-0.325
					KNPSKVIKS	-0.355
					SDKGKNPSK	-0.358
					NPSKVIKSY	-0.384
					ASDKGKNPS	-0.392
					TASDKGKNP	-0.395
					KGKNPSKVI	-0.401
					NTASDKGKN	-0.433
					DKGKNPSKV	-0.488
139	214	76	-1.30568	NENVNEKCKFTLPPLKIHECNISCPNPLPFREYKPQTEGVSNLVGLPNNICLQKTSNQILKPKLISYTLPVVGQSG	NPLPFREYK	0.23
					LKIHECNIS	0.195
					PNPLPFREY	0.187
					PLKIHECNI	0.14
					PLPFREYKP	0.13
					CPNPLPFRE	0.095
					SYTLPVVGQ	0.084
					ISYTLPVVG	0.076
					KPQTEGVSN	0.069
					KIHECNISC	0.068
					YKPQTEGVS	0.065
					KCKFTLPPL	0.059
					GLPNNICLQ	0.057
					LVGLPNNIC	0.055
					VGLPNNICL	0.054
					SNLVGLPNN	0.047
					VSNLVGLPN	0.043
					PQTEGVSNL	0.042
					CKFTLPPLK	0.04
					SCPNPLPFR	0.028
					YTLPVVGQS	0.025
					NLVGLPNNI	0.017
					LPFREYKPQ	-0.004
					LPNNICLQK	-0.007
					PPLKIHECN	-0.008
					PKLISYTLP	-0.008
					GVSNLVGLP	-0.01
					LPPLKIHEC	-0.014
					LISYTLPVV	-0.015
					EYKPQTEGV	-0.053
					ISCPNPLPF	-0.061
					QTEGVSNLV	-0.061
					IHECNISCP	-0.074
					TLPVVGQSG	-0.084
					NENVNEKCK	-0.086
					KPKLISYTL	-0.088
					ECNISCPNP	-0.093
					TLPPLKIHE	-0.097
					EKCKFTLPP	-0.1
					TEGVSNLVG	-0.1
					HECNISCPN	-0.102
					KLISYTLPV	-0.106
					EGVSNLVGL	-0.115
					FTLPPLKIH	-0.14
					NISCPNPLP	-0.141
					KFTLPPLKI	-0.155
					PNNICLQKT	-0.155
					QKTSNQILK	-0.163
					PFREYKPQT	-0.166
					KTSNQILKP	-0.183
					TSNQILKPK	-0.188
					NEKCKFTLP	-0.198
					CNISCPNPL	-0.198
					LQKTSNQIL	-0.218

REYKPQTEG	-0.247
LKPKLISYT	-0.248
SNQILKPKL	-0.253
FREYKPQTE	-0.267
ENVNEKCKF	-0.27
NVNEKCKFT	-0.276
ILKPKLISY	-0.286
NNICLQKTS	-0.29
VNEKCKFTL	-0.319
QILKPKLIS	-0.366
NQILKPKLI	-0.377
NICLQKTSN	-0.408
ICLQKTSNQ	-0.437
CLQKTSNQI	-0.446
