# Supplementary figures and images for: Design and Preliminary Immunogenicity Evaluation of Nipah Virus Glycoprotein G Epitope-Based Peptide Vaccine in Mice
Source: Vaccines (Basel). 2025 Apr 18;13(4):428. doi: 10.3390/vaccines13040428 (PMC12031491; doi:10.3390/vaccines13040428)

Figure S1. Schematic diagram of NiV-G peptide design in all groups

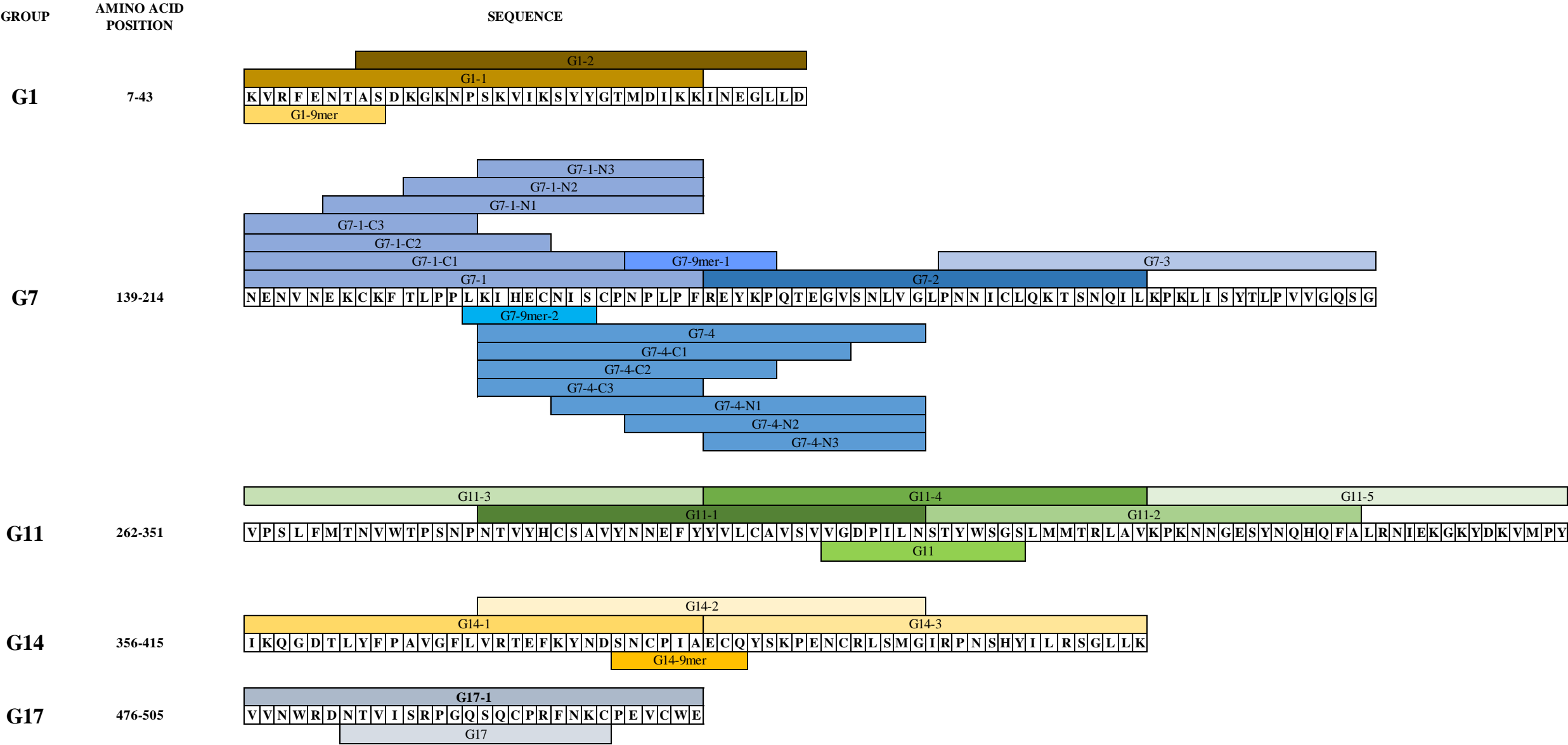

Supplement: Supplementary file 1 [file vaccines-13-00428-s001.zip › Supplementary Figure S1. NiVG peptide design schematic diagram_updated.pdf]

**Figure S2.** Immunogenicity evaluation of related truncated NiV-G G7-1 and G7-4.

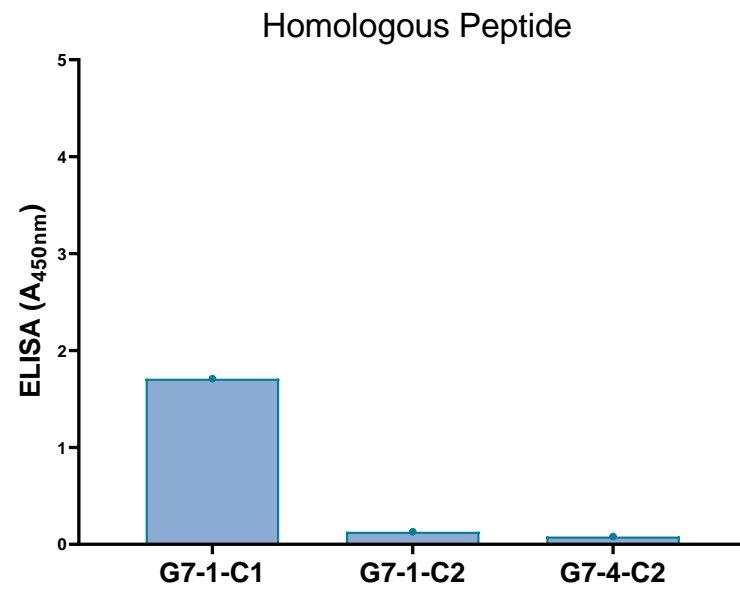

(a.)

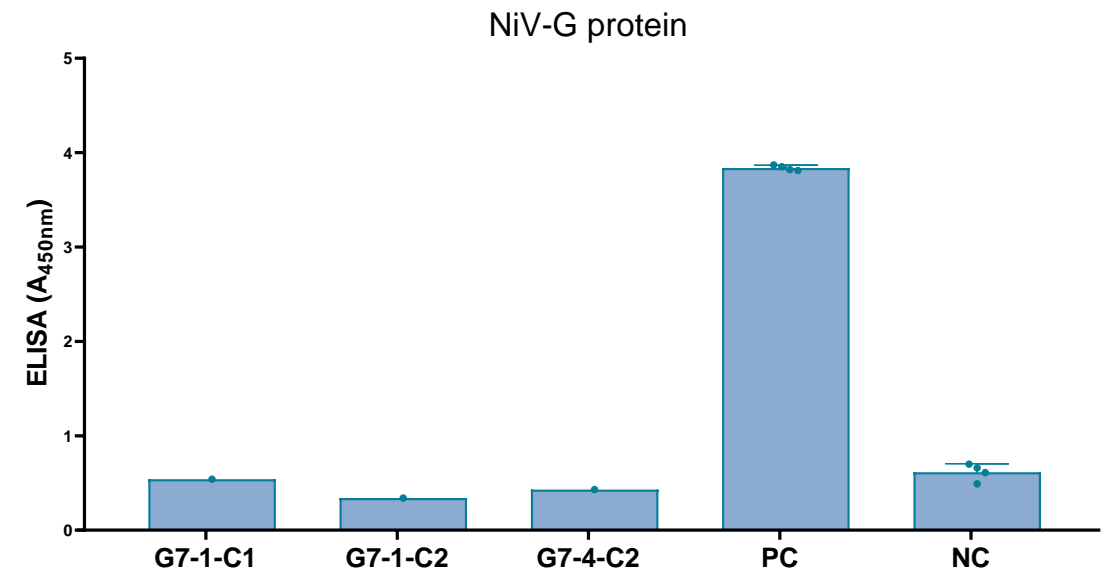

(b.)

Supplement: Supplementary file 1 [file vaccines-13-00428-s001.zip › Supplementary Figure S2. Immunogenicity of truncated NiV-G peptide candidates_updated.pdf]
